# Multi-omics analysis: Paving the path toward achieving precision medicine in cancer treatment and immuno-oncology

**DOI:** 10.3389/fmolb.2022.962743

**Published:** 2022-10-11

**Authors:** Virgile Raufaste-Cazavieille, Raoul Santiago, Arnaud Droit

**Affiliations:** ^1^ CHU de Québec Research Center, Université Laval, Québec, QC, Canada; ^2^ Division of Pediatric Hematology-Oncology, Centre Hospitalier Universitaire de L’Université Laval, Charles Bruneau Cancer Center, Québec, QC, Canada

**Keywords:** multi-omics, machine learning, immunology, immunomics, microbiome, cancer, precision medicine

## Abstract

The acceleration of large-scale sequencing and the progress in high-throughput computational analyses, defined as omics, was a hallmark for the comprehension of the biological processes in human health and diseases. In cancerology, the omics approach, initiated by genomics and transcriptomics studies, has revealed an incredible complexity with unsuspected molecular diversity within a same tumor type as well as spatial and temporal heterogeneity of tumors. The integration of multiple biological layers of omics studies brought oncology to a new paradigm, from tumor site classification to pan-cancer molecular classification, offering new therapeutic opportunities for precision medicine. In this review, we will provide a comprehensive overview of the latest innovations for multi-omics integration in oncology and summarize the largest multi-omics dataset available for adult and pediatric cancers. We will present multi-omics techniques for characterizing cancer biology and show how multi-omics data can be combined with clinical data for the identification of prognostic and treatment-specific biomarkers, opening the way to personalized therapy. To conclude, we will detail the newest strategies for dissecting the tumor immune environment and host–tumor interaction. We will explore the advances in immunomics and microbiomics for biomarker identification to guide therapeutic decision in immuno-oncology.

## Introduction

Cancer is an important cause of death worldwide, even if its mortality has declined during the last decade (Santucci et al., 2020). The International Agency for Research on Cancer (IARC) GLOBOCAN cancer statistics predicted an increase of 50% for the cancer incidence and an increase of 62.5% of the mortality from now to 2040 worldwide (Ferlay et al., 2020). There is an urgent need to better cancer survival rate; however, to cure this disease, we first need to understand its underlying mechanisms.

Until recently, cancer was widely considered as organ dependent and characterized by the site of apparition of the tumor. This hypothesis was slowly abandoned due to the heterogeneity present between two patients with a similar tumor (Dagogo-Jack and Shaw, 2018) that was identified through the emergence and democratization of next-generation sequencing (NGS). NGS allows the generation of a large amount of data in a short period of time, marking the beginning of a new era for cancer research: the genomics era (Knox, 2010; Lakshmanan et al., 2020). This led to a new characterization of cancer, not based on the tumor site but founded on molecular classification (pan-classification) (Hoadley et al., 2014; Campbell et al., 2018). Following the expansion of genomics, the exploration of the other layers of cancer biology started. The apparition of different omics such as transcriptomics, epigenomics, and proteomics allowed the possibility to understand the underlying complexity of tumors (Alyass et al., 2015). The dawn of omics studies led to the discovery of further complexity with cancer presenting intra-tumoral heterogeneity at a cellular level (Dagogo-Jack and Shaw, 2018). The study of these new omics highlighted an unsuspected complexity inside the tumor architecture but also furthered our understanding of interactions between cancer and its environment (gene-environment, microenvironmental interaction, and immune system interaction) (McAllister et al., 2017; Zhou et al., 2017; Gonzalez et al., 2018; Barriga et al., 2019).

The use of single omics, such as genomics and transcriptomics, uncovered many driver genes to better comprehend the genomic landscape of cancer. Interestingly, some of these studies revealed the wide complementarity of genomics and transcriptomics, many of the driver genes being identified by either one or the other modality (Wong et al., 2020; Berlanga et al., 2022). This fosters the need of combining the interconnected biological elements of cancer to move from single-omics to multi-omics analysis. The multi-omics integration is defined by the modelization of more than one biological element in order to characterize biological systems in its globality at the phenomenological level (de Anda-Jáuregui and Hernández-Lemus, 2020). The purpose of doing this is to look at how the different biological layers of the cells interact with each other, leading to the creation of an interconnected network highlighting the underlying complexity of cancer. Data integration in cancer have three main goals: understanding the molecular mechanism of cancer, clustering disease samples, and predicting an outcome (survival or therapy efficacy) (Hiley et al., 2014; Jamal-Hanjani et al., 2014; Tebani et al., 2016; Sharifi-Noghabi et al., 2019). Computational methods were needed to integrate the large diversity of data prompting the development of new algorithms to overcome the intricacies of multi-omics integration.

To this day, even with the new information that multi-omics approaches can bring, understanding how cancer develops and maintains is puzzling. Indeed, cancer cells interact with many different components including the host immune system (Witkowski et al., 2020). These interactions define the tumor immune microenvironment (TiME) that can be beneficial or detrimental to cancer cells, leaning toward more tolerogenicity or immunogenicity (Iwai et al., 2002; Garrido and Aptsiauri, 2019; Gou et al., 2020; Tang et al., 2020). To understand the TiME provides insights on how the cancer cells hijack the immune system to survive, but also might predict if a tumor is likely to respond to immunotherapy by immune checkpoint blockade (ICB) (Riaz et al., 2017). Indeed, biomarkers derived from the study of TiME appeared to be helpful to anticipate cancer sensitivity to immunotherapy (Goswami et al., 2020). Immunomics, the field of omics-based analysis that aims to describe the reaction of the immune system to another biological component (pathogen or cancer), can be used to analyze the interactions existing between the host immune system and the cancer cells and how it leads to immune recognition or immune ignorance (Arnaout et al., 2021). However, to fully depict the interconnection that exists between the tumor and the immune system, other players need to be taken into the equation. Indeed, it has been shown that the microbiome can influence the sensitivity of cancers to ICB, suggesting an interplay between the host microbiota and the immune constitution of tumor microenvironment (Baruch et al., 2021). Immunomics and microbiomics are two novel components that should be included into multi-omics models to integrate the tumor/host interaction in cancer complexity.

**FIGURE 1:**
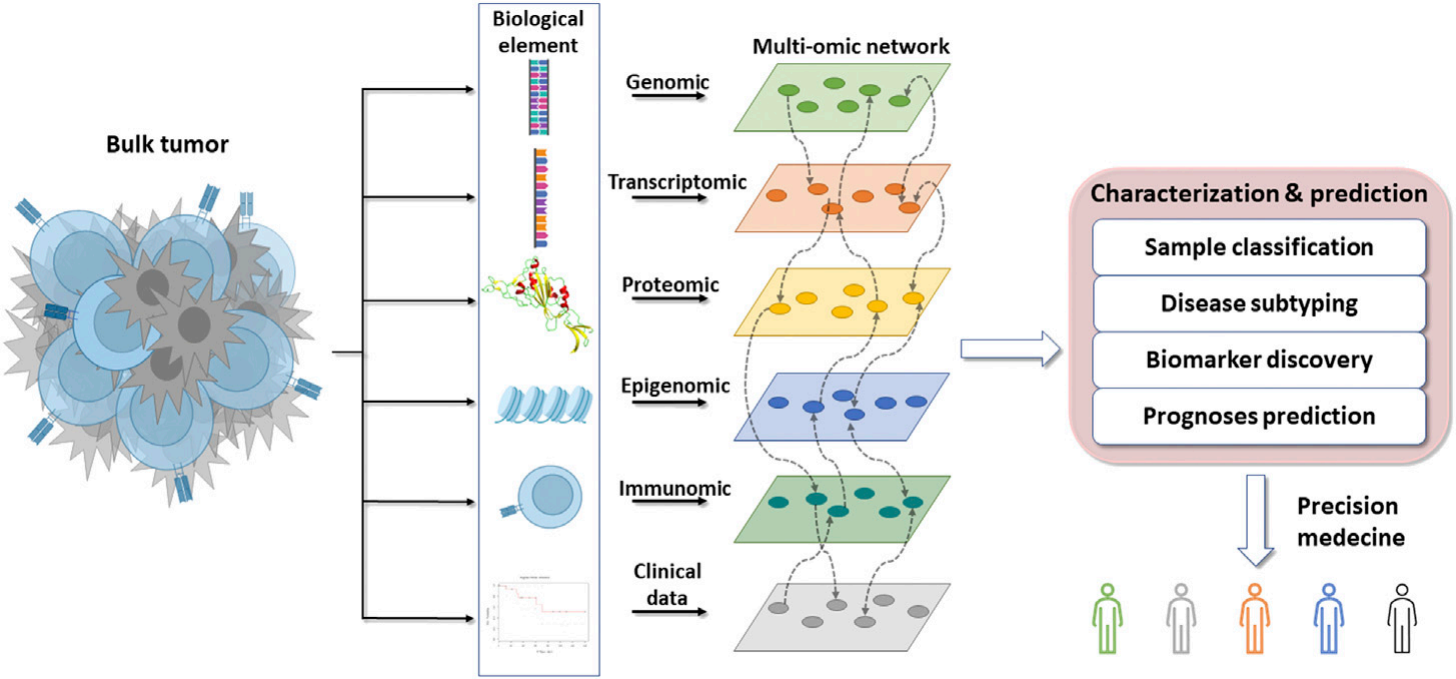
Overflow of the omics integration for precision medicine. The bulk tumor is composed of many different biological elements that can be classified and used to find novel interactions between interconnected elements. These interactions can be used to molecularly characterize and subtype cancers with the aim of anticipating their clinical evolution and treatment sensitivity to promote precision medicine.

In the first part, due to the growing interest in multi-omics, this review will address the challenges raised by omics and how to overcome them. In the second part, we will provide an overview of different databases that are useful in cancer research. In the third part, this review will attempt to illustrate how to integrate mutli-omics to clarify cancer complexity, and how the use of machine learning can help to predict survival and treatment response. Finally, we will give an overview of new methods to decipher the interaction between the host and the cancer cells and how it can bring opportunity for personalized therapy (Figure 1).

## Challenges

To integrate the massive data flow generated through the different biological elements explored by NGS, innovative computational tools are needed for diminishing the data dimension, then making them manipulable by human hands. These tools allow researchers to fulfill the gap of missing knowledges and contribute to the discovery of novel biomarkers, deciphering the complexity of cancers (Subramanian et al., 2020; Poirion et al., 2021; Wörheide et al., 2021; Zeng et al., 2021). Despite the progresses in these new instruments, multiple challenges remain.

One of these challenges is the heterogeneity that exists across the biological layers. It may differ in type, with numerical or categorical features, discrete or continuous variables with different ranges. The number of features can also vary between the different data sources, creating novel struggles to consider. Another burden, operating during sample collection, are missing values, setting up uninterpretable elements difficult to handle by some algorithms. Also, processing outliers and highly correlated variables might be burdensome to integrate as they might be irrelevant, create noise, or overfeed a system (Mirza et al., 2019; Song et al., 2020a).

To face these challenges, different tools have been created that we will summarize (Canzler et al., 2020; Nicora et al., 2020; Subramanian et al., 2020). Two main types of algorithms can be distinguished: 1) the “exploratory matrix” and 2) the “probabilistic matrix”. The first one is based on the matrix and its result. It performs best with a well-defined matrix, can handle outliers, but is less accurate when data are missing (Devarajan, 2008; Chauvel et al., 2020; Hamamoto et al., 2022). The probabilistic matrix utilizes probabilistic formulas (such as Gaussian and Bayesian). These algorithms shine when the dataset is incomplete, with missing values, but lose accuracy with outliers (Needham et al., 2007; Yuan et al., 2021; Chu et al., 2022). This approach aims to reduce the size of the matrix to identify patterns within the dataset, allowing for powerful classification models (Table 1).

**TABLE 1:** Description of different tools for multi-omics integration with their application and their major strength and limits.

Method	Principle	Aim	Omics element	Pros	Cons
JIVE	Matrix factorization	Disease subtyping, systemic knowledge, module detection	Genomics and epigenomics	Integrate large amount of data	Sensitive to outliers and missing values
NMF		Disease subtyping, module detection, biomarker discovery	Genomics and epigenomics	Filtering weak signal. Integrate large amount of data. Detection of cluster of small size	Time and memory consuming. Underperforming on missing values
nNMF					
jNMF					
intNMF					
SLIDE		Disease subtyping, module detection, biomarker discovery	Genomics, epigenomics and proteomics	Integrate large amount of data	Underperforming with missing values. Optimum solution is not guaranteed
MALA	Logic data mining	Sample classification	Genomics and transcriptomics	Works well on experimental data. Integrate large amount of data	Phenotype number must be delivered with data. Sensitive to missing values
iCluster	Gaussian latent variable model		Genomics, epigenomics and transcriptomics		Needs to test a large amount of solution to find the most relevant
iCluster+	Generalized linear regression	Disease subtyping	Genomics, transcriptomics, proteomics and epigenomics	Handle missing values	No evaluation of statistical significance for selected features
iClusterBayes	Bayesian integrative clustering	Biomarker discovery	Genomics, transcriptomics, and epigenomics	Good performance in the presence of explicative data	Underperform with outliers
MOFA	Bayesian factor analysis	Biomarker discovery, systemic knowledge	Proteomics, metabolomics and lipidomics	Handle well missing values	Linear model can miss linear relation
MOFA+			Genomics and epigenomics	The use of continuous learning enabling MOFA to recover different trajectory	Need of multi-modal measurement for the same set of cells

**JIVE**: joint and individual variation explained.

**(n, j, int) NMF**: (network, joint, integrative) non-negative matrix factorization.

**SLIDE**: structural learning and integrative decomposition.

**MALA**: micro array logic analyzer.

**MOFA**: multi-omics factor analysis.

### Exploratory matrix

Starting with exploratory matrices, herein is a brief overview of two algorithms using this approach: matrix factorization and logic data mining. These methods would be preferred for well-defined exhaustive datasets containing extreme values that need to be considered.

Matrix factorization: joint and individual variation explained (JIVE), non-negative factorization (NMF), and structural learning and integrative decomposition (SLIDE) are some of the tools based on this approach. This algorithm uses a system of multiplication between columns and rows on a dataset decomposed in multiple matrices of smaller dimension. Each matrices have n columns, referring to n common objects, that will be multiplied by m rows, referring to other common objects. The matrix can contain different biological elements, that is, gene expression and miRNA measurement. The strength of matrix factorization is to cluster different matrices together but requires a dataset with scarce missing values (Brunet et al., 2004; Devarajan, 2008; Lock et al., 2013; Pierre-Jean et al., 2022).

Logic data mining is a multistep procedure: 1) feature binarization, that assigns a threshold to convert each feature in binary values (0 or 1); 2) feature selection, that uses a machine learning approach to select a subset of data with relevant features introduced in a model; 3) extraction of logic formulas to build the final classification model. As an example, microarray logic analyzer (MALA) is based on this approach and has been used to identify overexpressed interrelated genes and proteins involved in a common pathway (Bertolazzi et al., 2008; Wang and éditeur, 2009; Weitschek et al., 2012).

### Probabilistic matrix

We will now detail four types of algorithms to better conceptualize the probabilistic matrix approach. These models follow the principles of Gaussian probability, linear regression, or Bayesian statistics and are very useful to troubleshoot datasets with missing values.

Gaussian latent variable model uses a probabilistic matrix starting with the dimension reduction of the dataset. The matrix composed of N columns and D rows will be decreased to a matrix with a lower dimensionality on D. D will be reduced in Q most relevant data. Relevant data will then be extracted to identify a pattern explaining the results. Because the algorithm is based on Gaussian probability, dataset with a Gaussian distribution perform better with this instrument than expression profiles near 0. Lower outliers close to the null value may cause misinterpretation during the analysis. However, missing values are well managed by the algorithm. A tool using this algorithm, iCluster, will be further explained in the next chapter (Li and Chen, 2016).

Generalized linear regression is one of the subcategories found in the generalized linear model. This algorithm is composed of three components: 1) random component; 2) systematic component; and 3) link function. The first step is the matrix generation, then a linear regression will be applied to the matrix finding the best possible linear relation to fit the expected values. The systematic components are distributed on the line while random components are outside of the line. The link function will identify a relationship between the linear predictor and the distribution of the random components. It aims to explain why some values are following a linear repartition while others are not. This model performs well when using data that are already explained on unexplained data. The accuracy might, however, be hindered by outliers that may introduce misinterpretation. This model is used in the icluster + tools (Mo et al., 2013; Song et al., 2022).

Bayesian integrative clustering: the objective of this approach is to capture the major variations of multiple omics datasets, using a reduction of the high-dimensional space to a low-dimensional subspace. This could be vulgarized into a compaction of the data. The algorithm will extract the principal variations of the datasets to integrate matrices of different dimensions to a single matrix called *Z* with *n* x *k* dimensions, following the rule of multivariate normal distribution. A joint integrative clustering will capture relevant features to individualize distinct clusters across omics datasets. The use of Baye’s theorem enables the analysis of factors with various distributions and correlation among datasets. The probabilistic model is also permissive of missing values (Needham et al., 2007; Fang et al., 2018; Mo et al., 2018).

Bayesian factor analysis: this is a key component of the multi-omics factor analysis (MOFA) family tools used for multi-omics data integration. This unsupervised method infers principal component-based factors to decompose each matrix of M different omics components. The matrices will be transformed into a Z matrix of factors for each sample and M weight (W) matrices with features in rows and factors in columns, for each omics element. Downstream analysis will identify inference across Z and W matrices. Also, because the algorithm is based on a Bayesian framework, the distributions of the data are placed on the unobserved variables of the models and the algorithm will keep running until all the data have been characterized (Needham et al., 2007; Argelaguet et al., 2018; Min et al., 2018).

To finish, despite the numerous tools that are now available, the perfect tool does not exist. The continual development of computational methods necessitates systematic evaluation (benchmarking) of the omics data analyses tools and methods (Mangul et al., 2019). The major issue on this benchmarking is the lack of “gold standard” datasets, providing unbiased ground truth. This lack of gold standard hinders the possibility to establish generalizable benchmarks to test novel complex software (Mangul et al., 2019; Weber, 2019; Marx, 2020). Different notable benchmarks are available comparing: multi-omics and multi-view clustering algorithms (Rappoport and Shamir, 2018), multi-omics dimensionality reduction (Cantini et al., 2021), multi-omics for cancer subtyping (Duan et al., 2021), and multi-omics survival prediction methods (Herrmann et al., 2021). Regardless of the use of the same dataset for comparison, the results obtained are not necessarily reproducible on another database and cannot be apply to the integration of omics that were not included in the initial dataset. The lack of a gold standard dataset impairs the success of the benchmarking (Krassowski et al., 2020).

## Multi-omics database

The expansion and acceleration of NGS have generated a tremendous amount of data. In a collaborative effort, extensive omics databases have been created to stock and make those data publicly accessible to researchers, allowing for large meta-analysis for a tumor category or across cancer types. These datasets can host the sequencing output of different biological elements from bulk tumor or single-cell sequencing as well as clinical and treatment information. We will provide a summary of the main databases that are available for cancer research and detail the characteristics of each library with tumor types included and the biological and clinical contents provided (Table 2).

**TABLE 2:** Publicly available cancer databases with their main characteristics.

Database	Multi-omics data available	Disease	References
**TCGA** (The Cancer Genome Atlas)	20,000 individual tumor samples	Cancers	(Weinstein et al., 2013; Tomczak et al., 2015)
	RNA-Seq, DNA-Seq, miRNA-Seq, SNV, CNV, DNA methylation, and RPPA, clinical data (treatment received and response to treatment), histological data		
**TCIA** (The Cancer Immunome Database)	8,000 tumor samples	Cancers	Charoentong et al. (2017)
	Genomic immune-related gene set, immune infiltrate, neoantigens, cancer antigens, HLA types, and tumor heterogeneity		
**ICGC** (International Cancer Genomics Consortium)	20,383 samples listed	Cancers	(Hudson et al., 2010; Australian Pancreatic Cancer Genome InitiativeBailey et al., 2016; Thompson et al., 2018)
	Somatic and germline whole genome sequencing, genomic variation data		
**METABRIC** (Molecular Taxonomy of Breast Cancer International Consortium)	2,503 breast tumor samples	Breast cancers	Curtis et al. (2012)
	Clinical traits, gene expression, SNP, and CNV.		
**TARGET** (Therapeutically Applicable Research to Generate Effective Treatments)	24 different types of pediatric cancers	Pediatric cancers	(Ma et al., 2018; Rajbhandari et al., 2018)
	Gene expression, miRNA expression, CNV, and DNA-seq data		
**CRI** (Cancer Research Institute) **iAtlas**	10,000 tumors samples	Cancers	(Thorsson et al., 2018; Eddy et al., 2020)
	Clinical data (immunotherapy responses and clinical phenotypes), genomics, immunomodulatory genes, neoantigens load		

**DNA-seq:** deoxyribo nucleic acid-sequencing.

**RNA-seq:** ribonucleic acid-sequencing.

**miRNA-seq:** micro ribonucleic acid-sequencing.

**SNV:** single nucleotide variant.

**CNV:** copy number variation.

**SNP:** single nucleotide polymorphisms.

**DNA, methylation:** deoxyribo nucleic acid methylation.

**RPPA:** reverse-phase proteomic arrays.

**miRNA, expression:** micro ribonucleic expression.

**HLA:** human leukocyte antigen.

The Cancer Genome Atlas (TCGA) is the largest pan-cancer multi-omics database with clinical annotation allowing for large meta-analysis. TCGA is widely used by the research community, promoting new discoveries on tumor biology, evolution, and treatment specific biomarkers validated on meta-analysis across cancer types (Weinstein et al., 2013; Tomczak et al., 2015).

The Cancer Immunome Database (TCIA) is a new database of immunogenic analysis of NGS data from 20 different types of solid tumors derived from TCGA database. This database has served for the elaboration of a pan-cancer immunogenomic classification for checkpoint blockade sensitivity (Charoentong et al., 2017).

The International Cancer Genomics Consortium (ICGC) is the most ambitious biomedical efforts research since the human genome project. This database has permitted novel observation of cancer biology through the whole genome annotated alterations from 2,800 samples (Hudson et al., 2010; Australian Pancreatic Cancer Genome InitiativeBailey et al., 2016; Thompson et al., 2018).

The Molecular Taxonomy of Breast Cancer International Consortium (METABRIC) contains genomic data from breast cancers. This database helped improving the classification and subtyping of breast tumors and already led to the discovery of 10 subgroups (Curtis et al., 2012).

Therapeutically Applicable Research to Generate Effective Treatments (TARGET) is a clinically annotate database hosting genomics and transcriptomics data from pediatric tumors. This dataset empowered the description of the driving process of childhood cancers, and was used, for example, for characterizing the immune environment of pediatric cancers and its prognostic impact (Ma et al., 2018; Rajbhandari et al., 2018; Sherif et al., 2021).

Cancer Research Institute (CRI) iAtlas is a database platform allowing the study of interaction between tumors and immune microenvironment, granting the possibility to explore immune response across genomics and clinical phenotypes. iAtlas (first version) allows researchers to explore these readouts and the relation between tumor types and immune response (Thorsson et al., 2018). CRI iAtlas now shares information about immunotherapy response useful for biomarker identification (Eddy et al., 2020).

The dramatic acceleration in data production and the policy for data sharing have increased the comprehension of cancer complexity and fostered the integration of interrelated biological elements of omics analysis in more and more complex computational models that we will show in detail in the following.

## Multi-omics for greater accuracy

### Multi-omics for a better classification of cancer types

Tumor heterogeneity remains a hurdle to understand tumor biology. For example, for a same tumor type, there is a wide variation in clinical evolution across population of patients, highlighting differences in tumor cells to progress or mutate (Lagoa et al., 2020; Wu et al., 2020). These differences are barriers to develop efficient therapies (Marusyk et al., 2012; Cyll et al., 2017; Marusyk et al., 2020). Cancer is relatively easy to classify as the classification is based on the histotype and site of origin of the tumor. However, the classification system has increased in complexity in the last decades with the introduction of genomics and molecular features to better account for the clinical evolution and foster the identification of histologic groups (Carbone, 2020). Therefore, omics data can be used to individualize tumor types. In this aim, a variety of bioinformatic tools have been developed to refine and accelerate sample classification.

One of these tools that allows the sample classification is a microarray logic analyzer (MALA). MALA is based on logic data mining algorithm. Its follows a three-step process: (Santucci et al., 2020) discrete cluster analysis, (Ferlay et al., 2020) selection of the most relevant (cluster of) genes, and (Dagogo-Jack and Shaw, 2018) logical formulas characterizing the samples (Weitschek et al., 2012). In the context of cancer research, a comparative study came out with the purpose to evaluate which tools could be the most accurate in treating multilevel omics data. MALA with sparse canonical correlation analysis (SCCA) and non-negative matrix factorization (NMF) were compared. When using the experimental data, MALA performed the best sample classification compared to the others. However, on a larger data set of simulated data, the efficiency of the model decreased (Pucher et al., 2019).

### Multi-omics for cancer subtyping

To better understand cancer, researchers need to characterize the different cancer subtypes. Subtyping cancer is more complicated and remains one of the major challenges in cancer research. The identification of subtypes can provide an understanding of the underlying molecular mechanisms and thereby help design precise treatment strategies for efficient cancer management. Contrary to classification that is more histologic, the subtypes are influenced by oncogenic alterations and/or modifications in the gene/protein expression (Anderson et al., 2021). Molecular classification has partially elucidated tumor heterogeneity, however, different subtypes can be identified depending on different layers of biological elements: genomics, alterations, gene and protein expression profile as well as cellular composition (Skoulidis and Heymach, 2019). The main challenge is to be able to accurately analyze the large amount of data and to determine and isolate predictive patterns. Multi-omics provides a powerful approach to process, treat, and characterize the large quantity of data required for cancer subtyping (Menyhárt and Győrffy, 2021).

Cancer subtyping can be efficiently addressed by matrix factorization, such as joint and individual variation explained (JIVE) algorithm. This tool aims to individualize two types of structures: joint and individual structures. The former is the biological patterns of the samples that are shared between the different component types (*i.e*., gene expression and miRNA), while the latter is intrinsic of one component and unrelated to others (Lock et al., 2013). One structure can interfere in finding a signal in the second structure and *vice versa*. JIVE was developed to distinguish these possible interfering effects by decomposing the datasets into a sum of three terms: (Santucci et al., 2020) low-rank approximation capturing joint variation across the biological components, (Ferlay et al., 2020) low-rank approximation capturing structured variation specific to a given component, and (Dagogo-Jack and Shaw, 2018) residual noise. It explores the information provided by a specific biological data type or by the interaction between several data for subtyping. A logical weakness of this method is its sensitivity to outliers. JIVE algorithm was tested on 234 glioblastoma samples, with an input of miRNA and gene expression matrices. It showed that the gene expression introduced more structured variation than miRNA and that joint structure variation between the gene expression and miRNA was more accurate to classify the biological subgroups. Overall, the multi-component integration improved the subclassification of glioblastoma samples.

In the optic of diseases subtyping, a growing diversity of tools based on matrix factorization were developed. As an example, non-negative factorization (NMF) consists in multiplication between the columns and the rows of a matrix. The input data sets are formed by a matrix called *A*, composed of *N* genes and *M* samples. Genes that regulate the expression of downstream genes will be identified and labeled *k*. In the second step, the matrix *A* will be split in two matrices: *W* composed of the *N* genes and *k*, and *H* composed of *k* and *M* samples. Finally, *W* and *H* matrices will be combined in a new matrix. The advantage of the NMF technics is to easily cluster gene and samples, but this is a time and memory consuming tool that does not handle negative input and is not designed to integrate multilayer components (Lee and Seung, 1999; Brunet et al., 2004; Zhang et al., 2012; Yang and Michailidis, 2015).

Briefly, NMF extensions were implemented to allow multi-omics profiling. Multi-omics integration with jNMF was compared to single omics to class clinical data from TCGA into different subgroups and outperformed the single element model. Moreover, the number of groups will depend on the clinical data used (Zhang et al., 2012). A second major update was intNMF, while jNMF only considers homogenous effects in *WHI* and intNMF considers heterogenous effects (Yang and Michailidis, 2015). As an example, breast cancer subtyping with multi-omics data integration of five biological components by intNMF refined the subclassification from four classical subsets to six unique clusters (Chalise and Fridley, 2017).

The last update of NMF is called network-based integrative (nNMF). nNMF involves a two-step process with network generation and integration of the network. To generate the network, a consensus matrix is built with a binary value attributed to each sample to reflect the connectivity between samples. Successive cycles are performed attributing a new binary value for every novel entry until the matrix is stabilized. The mean of the consensus matrix is made for each iterative cycle until the last cycle. This generates a consensus matrix for each data type integrated. The larger elements of the consensus matrix reflect the higher similarity between samples. After the networks are generated, samples can be considered as vertices and the consensus values as the edges. The network integration is based on the message passing theory (updating and combining network) that is processed on two ways: strong signals present in any data are conserved and consistent signals in multiple data are added up during an iterative process. Weak signals disappear while filtering out the noise. An advantage of nNMF is its ability to detect true cluster of small size with high reliability. Clinical application of nNMF demonstrated the capacity to establish novel clusters on head and neck squamous cell carcinoma, glioblastoma, and low-grade glioma data sets, suggesting a new comprehensive subtyping that eliminates previously unclassified samples (Chalise et al., 2020).

NMF and its extensions are based on matrix factorization algorithm that are efficient tools to treat a massive amount of data but are underperforming with missing values. To complement this part, iCluster family tools are good alternatives. iCluster is based on probabilistic matrices algorithm and iCluster is based on a Gaussian latent variable model. The basic concept of iCluster is to jointly estimate the link between the data with a dimension reduction principle: data and features are clustered together to maximize the correlation between data types (Shen et al., 2009). iCluster was used to classify novel subtypes of esophageal cancers based on genomics, epigenomics, and transcriptomics data. The classification varied depending on the type of omics. Compared to previous classification, the samples were consistently classified into three groups with different biological traits and prognostic significance (Ma et al., 2021). It was also used in breast cancer to integrate DNA and RNA data leading to novel subtyping with noticeable clinical outcomes beyond classic expression profiling (Curtis et al., 2012).

The first extension of iCluster was iCluster+ (or iClusterplus), a model based on a generalized linear regression combined to the basic algorithm of iCluster. Compared to different models, iCluster + produces the best classification when integrating unknown datasets. It has been useful to construct two molecular subtypes and identified two core genes (*CNTN4* and *RFTN1*) for lung adenocarcinoma (Zhao et al., 2021). Two limitations to this tools can be pinpointed: it needs to test hundreds of values to tune the optimal solution parameters and there is no evaluation of statistical significance for a selected feature (Sathyanarayanan et al., 2020).

The most recent upgrade is iClusterBayes, which uses a Bayesian integrative clustering algorithm. The main advances of iClusterBayes are to overcome the limitations of iCluster + that were priorly exposed. This tool was tested on kidney cancer and glioblastoma and grants the possibility for tumor subtyping. However, iClusterBayes was not compared to another method, so that its capacity to discover novel subtypes could not be assessed (Mo et al., 2018).

SLIDE is a tool based on the concept of the multi-view of data, using a matrix factorization algorithm. SLIDE was developed in the continuity of JIVE, trying to investigate on shared and individual structure. Compared to JIVE, SLIDE allows the creation of partially shared scores in addition to the individual ones. SLIDE was able to upgrade breast cancer subtyping using gene, methylation, miRNA, and protein profiling (Gaynanova and Li, 2019).

The integration of multiple layers of interconnected biological elements through a wide palette of multi-omics tools available is a great opportunity to better classify biological-relevant cancer subtypes and to reduce unclassified samples. The choice of the algorithm should judiciously fit the characteristic of a given dataset to optimize the model performance.

## Biomarker discovery

The tumor biology is intimately related to disease evolution and treatment response (Marusyk et al., 2020). The assimilation of clinical data as additional features to feed multilayer integrated models enables association between molecular subtypes and clinical outcome. Discovering biomarkers associated with prognosis and treatment sensitivity/resistance is a keystone for risk-group classification and therapeutic decision. The identification of treatment specific biomarkers is also granting the opportunities to provide therapies tailored to the biological trait of a specific tumor, opening the path for precision medicine. Machine learning and deep learning models, trained on Kaplan–Meier derived survival data, are powerful classifiers to predict the clinical evolution. In this part, we aim to illustrate how multi-omics integration alongside machine and deep learning approaches might facilitate biomarker discovery and guide treatment decision.

### Application of multi-omics to biomarker discovery

Multi-omics tools have been developed to discover novel biomarkers in oncology. For example, jNMF allows biomarker discovery for prediction of drug response through pathway signature analysis. The identification of novel connections between tumor biology and drug response highlighted an association between BRAF inhibitors efficacy and *BRAF*/*MITF* overexpression in breast cancer (Fujita et al., 2018). Also, iClusterBayes demonstrated its ability to discover biomarkers, revealing the role of *MTAP*/*CDKN2A*/*2B* expression for PD-L1 blockade sensitivity with a proportional relation of Kaplan–Meier survival to the gene expression level (Mo et al., 2020).

Lemon-tree is another tool for multi-omics processing that runs a series of tasks that are self-contained step in the learning and clustering process. The workflow of the tools is as follow: ask biological question → preprocess data → clusterization → builds modules of cluster → compute score → results. Using this tool to discover biomarkers was operated on glioblastoma genes, looking at the amplification levels and copy-loss level of genes. The results show that genes that have copy number alteration (CNA) of glioblastoma oncogene *EGFR* and tumor suppressors *CDKN2A* and *PTEN*, but also novel candidates such as *KRIT1* and *PAOX* were assimilated with a worse prognosis (Bonnet et al., 2015). iProFun, is a method analyzing the “cascade effects” of the genes. It takes as input statistics associated with the data, aiming to detect the joint variation between each data. This study highlighted potential therapeutic candidates (*AKT1*, *KRT8*, and *MAP2*) in ovarian cancers. But it also demonstrated the role of *BIN2* in ovarian cancer, and how it can be a favorable survival outcome (Song et al., 2019). Finally, AMARETTO is used to identify driver genes by integrating genomics and epigenomics data. This is a three-step process, identifying candidate cancer driver genes, modeling effect on gene expression, and association of drivers with their targets. In other words, it creates clusters depending on driver genes and expression of the genes. AMARETTO was able to highlight different driver genes such as *GPX2* for smoking induced cancer (lung squamous cell carcinoma), but also identified *OAS2* and *TRIM22* as modulator of the immune response (Champion et al., 2018).

### Machine learning model for biomarker discovery

Machine learning are algorithms that can predict models using statistical methods based on training data. Algorithms can be trained in two ways: *via* supervised or unsupervised learning. Supervised learning relies on a labelled input dataset used to train and develop a function to predict the outcome on an experimental dataset. The algorithm attempts to find patterns relating the features to the given label. The second step is to integrate data test and to classify the validation data in the right label by following the identified patterns. The correlation between the given and predicted labels can be compared to assess the accuracy of the model. Unsupervised learning clusters the samples by identifying and regrouping different features following a similar pattern (Huang et al., 2015). The main difference of supervised learning is that the input data are not labelled. Machine learning approaches algorithms containing only one hidden layer.

A powerful tool developed for multi-omics integration is the artificial neural network (ANN). It can be used for machine learning and for deep learning. ANN is a simple approach to create an artificial model, composed of neurons organized in layers. This is composed of an input layer, hidden layer, and an output layer. The input layer corresponds to the dataset given to the algorithm and the output layer corresponds to the possible outputs of the algorithm, in this case, a classification of the data. The hidden layer is what defines the complexity of the algorithm, it represents possible pathways linking the input layer to the output layer. For the integration of multi-omics, different blocks, specific to each type of omics data, are created. Depending on the biological question, different machine learning algorithms can be preferred.

A vast diversity of algorithms are available for machine learning; random forest classifier and k-nearest neighbor are commonly supervised classifiers that are easily suitable for biomarker discovery. A random forest algorithm builds multiple decision trees that are randomly generated. Each decision tree provides a classification. The classification that is chosen in a majority of cases is used to classify each datapoint of the dataset (Tin Kam, 1995). K-nearest neighbor follows three main steps: 1) a reference dataset is clustered into the different classes that need to be distinguished, 2) the experimental data are integrated into the dataset, and 3) the experimental data are classified by calculating the distance to each defined class and classifying each datapoint in the nearest cluster (Fixt and Hodges, 2022).

These different approaches have been successfully applied to biological data to improve prognoses prediction. For example, ANN has already been used to analyze the survival in two breast cancer datasets. The model was able to predict the prognoses (favorable or unfavorable) and relapse probability (Chi et al., 2022). In the second example, machine learning approaches were compared to histopathological grading to classify and predict patients’ outcome in glioblastoma. In this test, a k-nearest neighbor-based ANN approach out scaled the histopathological grading for tumor classification and survival prediction. (Petalidis et al., 2008). In the third example, a random forest algorithm allowed the discovery of a novel prognostic biomarker in the Ewing Sarcoma. As an example, a lower Ki67 expression was associated to a better prognosis for the subset of samples with low-CD99 expression (Bühnemann et al., 2014). In the final example, a gene expression-based algorithm of k-nearest neighbor and random forest identified novel prognostic genes had increased the accuracy for the classification of the poorly defined group of soft tissue sarcomas. They were also able to annotate the samples in molecular subsets with potential therapeutic susceptibility (van IJzendoorn et al., 2019).

### Deep learning for treatment guiding

Deep learning approaches are a subset of machine learning that contains multiple hidden layers organized in a network allowing for progressive learning (LeCun et al., 2015; Bi et al., 2019). Deep learning can be deep neural network (DNN) or convolutional neural network (CNN). DNN is similar to ANN with an increased number of hidden layers, and CNN is a class of ANN used for imaging analysis (Simonyan and Zisserman, 2015; Szegedy et al., 2015; He et al., 2016; Chollet, 2017).

Survival analysis learning with multi-omics neural network (SALMON) attempts to aggregate and simplify the gene expression data to enable prognosis prediction. Deep learning approaches typically introduce an extensive number of parameters from a limited sample set that can introduce data overfitting rendering the models ineffective. In comparison, SALMON favorizes the use of eigengene matrices of gene co-expression modules to feed the model. This model was compared to other survival prognostic models on breast cancer datasets, including DeepSurv and random survival forest. SALMON predictor reached a higher survival concordance index than other methods and showed that the integration of multi-omics data combined with clinical and demographical features can increase the prediction performance. This algorithm was used to develop an age-specific prognostic score for breast cancer (Huang et al., 2019).

CNN is very useful for deconvolutional observations of histological images used to classify and predict cancer prognoses. CNN was successful to detect and to predict patient outcomes on haematoxylin-eosin-stained tumor tissue microarray with a higher accuracy than the visual prognostic prediction. For that, the image was cut into spots that were characterized with a pre-trained convolutional neural network (Bychkov et al., 2018). MesoNet is another tool developed to analyze larger images. To do so, the whole-slide images are subdivided in tiles that have a score assigned. Based on the score, the algorithm can select the most relevant tiles for the prediction. The cumulated score of the selected tiles informs the prediction of a patient’s overall survival (Courtiol et al., 2019). These tools perform well to analyze cancer heterogeneity. To finish, survival convolutional neural network (SCNN) is a CNN-based algorithm combined to an integration tool. This model identifies visual patterns from regions of interest isolated in biopsies and relates them to patient’s outcome to create a model. This comprehensive tool can also integrate genomics biomarkers. This technology was able to develop a better prognostic prediction tool compared to WHO genomics classification (Mobadersany et al., 2018).

DrugCell is a visible neural network for drug response prediction. The model is built with two branches. The first one modulates the hierarchical organization of molecular subsystems in human cells. Each subsystem, involving small protein complexes, is connected to larger pathways and to cellular functions assigned from a bank of artificial neurons. The input layer is the mutation status from genomics data and the output corresponds to the state of the whole cell based on the genotype. The second branch of the system is a CNN assessing the fingerprint of a drug. The output from the two branches of the model is combined in a single layer of neurons integrating the response of a genotype to a certain treatment. To test on the first side of the model, they used two large drug screening resources: the Cancer Therapeutics Response Portal (CTRP) v2 and the Genomics of Drug Sensitivity in Cancer (GDSC) database. Using these two resources, it covers 684 drugs and 1,235 cell lines. Each cell-line genotype was represented with a binary vector (mutated or non-mutated). The chemical structure of the drug was represented by an average of 81 activated bits in the Morgan fingerprint vector. Drug cell was trained to associate each genotype-drug paired with its corresponding drug–response curve. As a last point, drug cell was clinically tested stratify cancer patients depending on their response to treatment. They tested drug cell on clinical trial data from 221 estrogen receptor positive metastatic breast cancer patients and showed that the model was able to predict the response to mTOR and CDK4/6 inhibitors. Drug cell also includes a feature for predicting the sensitivity to drug combination (Kuenzi et al., 2020).

In a number of studies, DNN was used to predict prognoses. As an example, DeepSurv is a tool defined as a prognostic model. With the integration of time-dependent and treatment-sensitive survival data to the model, it was trained to provide treatment recommendations. DeepSurv was tested on four real-life datasets: Worcester Heart Attack Study (WHAS), the Study to Understand Prognoses Preferences Outcomes and Risks of Treatment (SUPPORT), the Molecular Taxonomy of Breast Cancer International Consortium (METABRIC), and Rotterdam & the German Breast Cancer Study Group (GBSG). DeepSurv takes input as the patient’s baseline data. The output is composed of one node estimating the log-risk function in the Cox model (estimation of the effect parameters without any consideration of the hazard function). As the second part, it uses a treatment recommender system. This system takes into account a group of patients assigned to a specific treatment group to predict the log-risk of a given treatment. Using the same base line hazard functions, it is possible to calculate the personal risk-ratio of prescribing one treatment and then, determine a treatment recommendation algorithm. To assess the possibility of a treatment recommendation system, DeepSurv was trained on the Rotterdam tumor bank to build the recommendation system that was then tested on GBSG. The treatment recommendation system demonstrated that, on a real clinical dataset, the patient’s survival would be increased by following the treatment recommendation of the algorithm. In comparison, a random survival forest-based recommendation system was not able to improve the patient’s outcome (Katzman et al., 2018). To conclude, DeepSurv provides strong modeling capabilities with the ability of guiding the therapeutic decision and bringing the clinician closer to computer-assisted technology for precision medicine in oncology.

Other examples of machine and deep learning models applicable for biomarker discovery are detailed in a recent review articles devoted to this subject (Nicora et al., 2020) (Zhu et al., 2020). So far, these powerful algorithms have been used to successfully develop models for prognosis and treatment response prediction. However, these cutting-edge technologies are not yet ready for personalized treatment recommendation in the perspective of prospective clinical implementation.

## New hope in omics: deciphering host–tumor interplay

Novel fields of interest have emerged to unravel host–tumor interactions (Hui, 1989) through the immunologic cancer response (Nisar et al., 2020) (Binnewies et al., 2018) and to understand how tumor cells can trick the host to hijack its defensive system and favor cancer immune evasion. The comprehension of the immune escape mechanisms opened the path to immune-mediated therapeutic opportunities. ICB are molecules that restore the immune system’s ability to recognize and eliminate tumor cells such as PD1/PDL1 (program cell-death protein one and its ligand) or CTLA4 (cytotoxic T-lymphocyte associated protein 4) inhibitors have revolutionized the treatment of some adult cancers (Nishino et al., 2017). However, not all the patients with a same disease will respond to these treatments and anticipating the patients who will be responders remains challenging. Indeed, ICB efficacy is highly dependent of the constitution of the tumor cell immunogenicity, the host immune recognition and, ultimately, the TiME (Bonaventura et al., 2019). The advances in NGS and the growing number of high-throughput tumor sequencing datasets alongside the constant improvement of computational analyzes opened the way to new disciplines like immunomics and microbiomics.

### ICB biomarker identification

The host-tumor interaction comprises a succession of interrelated steps facilitating or refraining an efficient immune recognition of tumor cells. These steps are dependent of the tumor biology, the host ability to recognize the tumor, and the mechanisms that will promote immune inflammation or immune evasion. Briefly, the somatic mutations of tumors result in the expression of neoantigens that can be exposed to and recognized by the host antigen presenting cells (APCs). This will activate an immune cascade responsible for the activation and recruitment of effector T cells in the tumor (Chen and Mellman, 2017). These primary steps determine the infiltration of the tumor by effector immune cells: the tumor infiltrated lymphocytes (TILs). The anti-tumor activity of TILs is influenced by the presence of stimulatory or inhibitory signaling. PDL1, expressed by tumor and immune cells, binds to the PD1 receptor on T cell and generates an inhibitory signal leading to T-cell exhaustion and loss of activity (Chen and Mellman, 2017). To be fully efficient, ICBs needs the tumor to be infiltrated by inflamed T cell rending possible to develop biomarkers of efficacy (Bonaventura et al., 2019).

The identification of biomarkers was first made possible by immunohistochemistry (IHC) assay that has the advantage of spatial annotation but is limited by the sparse number of features that can be assessed. A classification in three IHC immune states for tumor infiltrated lymphocytes (TILs) density is usually admitted with immune inflamed (or “hot”), immune excluded, and immune deserted (or “cold”) tumors (PMID: 30,179,157). Few biomarkers are so far broadly recognized. Some from the IHC like tumor infiltrated lymphocytes (TILs) density, mostly for CD8, and the protein expression of PD-L1 in the tumor. Other biomarkers derive from NGS test: tumor mutation burden (TMB) and the level of expression of interferon-gamma (*IFNγ*) or *INFγ* gene expression profile signature (Ayers et al., 2017; Nishino et al., 2017). These biomarkers are known to be independent predictors of response to ICB, however the predictive value of cumulative biomarkers remains partially solved, so that the integration of multilayers omics analysis for dissecting the immune environment through immunomics was brought to the fore.

### Immunomics for TiME profiling

Immunomics is the part of omics integration that aims to comprehend the host-tumor interaction and to profile the TiME. Integrative immunomics allows for quantitative, functional, spatial, and clonal annotation of the immune environment to comprehend the host/cancer interaction and discover biomarkers for ICB efficacy.

To integrate and study immune cell-host interactions, novel bioinformatic tools were developed. The quantification and enumeration of immune cell infiltrate can be deducted from bulk-tumor RNAseq data by deconvolution-based tools, such as MCP-counter (Becht et al., 2016) and CIBERSORT (Newman et al., 2019), or through gene expression scores (Danaher et al., 2017). It is also possible to use computational analyzes to infer the somatic mutations and the neoantigens presented by the tumor cells as well as the immunogenicity of these neoantigens, for example, the TMB assesses the mutational load of a tumor (Endris et al., 2019). The enumeration of intra-tumor T-cell and B-cell clonotype expansion, through T-cell receptor (TCR) and B-cell receptor (BCR) rearrangement, are particularly interesting as a surrogate of the immune reactivity potentially induced by the tumor. Different tools exist to extract the T-cell and B-cell clonotype repertoire from genomics or transcriptomics data, such as MiXCR (Bolotin et al., 2015) and immuneDB (Rosenfeld et al., 2018). These methods align VDJ sequences, quantify them, and sort them in distinct clonotypes. With the development of these tools, high-throughput NGS dataset can be analyzed through the spectre of immunomics and for discovering new biomarkers of ICB efficacy. We will, herein, present some of the latest studies of immunomics integration.

A pan-cancer analysis, from the publicly available TARGET dataset, aimed to characterize and classify immune subsets of pediatric solid cancers based on transcriptomics and survival data (Sherif et al., 2021). The gene expression profile and gene set enrichment analysis (ssGSEA) individualized six distinct immune groups with diagnostic and prognostic association. The study used the data from 408 samples from five pediatric cancers: neuroblastoma, osteosarcoma, and three kinds of renal cancers.

The immune infiltrate was first assessed by the immunologic constant of rejection (ICR) method (Thorsson et al., 2018) in three classes from low- to high-immune score. Kidney rhabdoid tumors had the higher immune score and Wilms tumor the lowest. Correlation clustering of ssGSEA-based immune signatures identified five main modules: interferon-gamma (IFN-G) and tumor growth factor beta (TGF-B) signaling, macrophages, lymphocytes, and wound healing. These five modules were used for the identification of six immune subtypes: T-cell helper 2 (Th2) dominant, inflammatory, immunologically quiet, wound healing dominant, macrophages dominant, and lymphocyte suppressed subtypes. Each immune subtype was composed by a specific immune infiltrate by CIBERSORT enumeration, that mirrored the immune signature.

Tumor types were unevenly distributed between the immune groups, and thus highlighting the association of the immune phenotype and the diagnosis. Furthermore, the immune subtyping was correlated with the prognosis, inflammatory subtype having the best clinical outcome while wound healing dominant subtype had the worse survival. The clinical impact was observed across cancers and within a same cancer type. This work demonstrates that access to NGS, well clinically annotated large tumor database, and immunogenomics integration help understand the interplay of host immune system and tumor cells. The immune landscape characterization can also improve tumor classification and prognostication as shown in this pediatric database.

A systematic meta-analysis of tumor and microenvironmental biomarkers for ICB sensitivity has investigated the relative impact of independent and combined biomarkers. An immunomics analysis from bulked-tumor transcriptomics and genomics was performed on a dataset from seven cancer types from 1,008 adult patients (CPI1000+) treated with immune checkpoint blockade (ICB) (Litchfield et al., 2021). The goal of this work was to select a large panel of biomarkers, aggregated by a systematic literature search, to assign a Z score for each of them and test their prediction impact for ICB efficacy in a large pan-cancer population. The different biomarkers explored the T-cell response, the mechanisms for immune evasion and infiltration, and the host factors.

In univariate analysis, clonal and total TMB and *CXCL9* expression, a CD8 attractive chemokine, were the strongest predictors for ICB response, followed by *CD8A* expression, T-cell inflamed *INFG* gene expression, and *CD274* expression. Due to the high prevalence of TMB in the literature, researchers decided to subdivide the somatic mutation by mutations fitting in an immunogenic signature. Some signatures like dinucleotide variants, that are source of amino-acid changes and generate immunogenic epitopes, ultraviolet (UV), or tobacco mutation signatures also came out as significant determinant of ICB response. In somatic copy number analysis, two copy number anomalies were identified as positive or negative predictor of response. Surprisingly, host factors like the loss of HLA heterogeneity or HLA subtypes did not show any impact on ICB sensitivity. They also performed single-cell RNA sequencing of a reactive CD8 TILs from patients to identify T-cell intrinsic markers of ICB sensitivity and highlighted *CXCL13* and *CCR5* gene involvement. The immune cell enumeration or BCR/TCR clonality assay were not assessed in this study.

A machine learning model for multivariate analysis including the significant determinant of response was a better predictor of ICB response than TMB alone in the initial cohort and three external independent validation datasets. Also, a two-parameter biomarker model combining clonal TMB and *CXCL9* expression had a better prediction accuracy than clonal TMB alone, yet inferior to the multivariate model. The integration of multiparameter biomarkers could explain approximatively 60% of the response to ICB in the different cancer types.

This study showed that multilayers integration of immunogenomics data and multiparameter models can improve the prediction of biomarker to anticipate the response to immunotherapy. It is open the opportunity to mechanistically solve host-tumor interaction and to understand how ICB remodel the tumor environment.

Others studies of multilayer immunomics analysis depicted the role of B cell in promoting ICB efficacy (Anagnostou et al., 2020; Helmink et al., 2020). In Helmink’s study, conducted on multiple cohorts of melanoma and renal cell carcinoma treated with ICB, for comparison between responders and non-responders, or immunotherapy naïve to test the prognostic impact of B-cell infiltrate. The input data comprised RNAseq, deconvolution immune cell enumeration, BCR clonality, single-cell RNA sequencing, and mass cytometry (CyTOF) for functional analysis and histological evaluation for spatial annotation. The gene expression profile showed an increase in the activation marker genes of B cells (*IFNG*, *MZB1*, *JCHAIN,* and *IGLL5*) in ICB responders. Using TRUST-algorithm for BCR clonotype enumeration, they observed an increase of clonal counts for heavy and light chains and in BCR diversity in responders. MCP-counter deconvolution algorithm also confirmed the enrichment of B cells in responders compared to non-responders. To confirm the role of B cell in ICB activity, they used IHC to assess B-cell density and to interrogate the spatial repartition of B cell and they performed functional study. The IHC confirmed an increase in B-cell density in responders and revealed a spatial organization in tertiary lymphoid structure (TLS) where CD20^+^ B cells colocalize with CD4^+^, CD8^+^, and FOXP3+ T cells. A previous study similarly showed a correlation between B-cell signature and increased the expression of *CD8A* and CD8^+^ T-cell infiltration (Griss et al., 2019). Functional characterization of tumor B cell by single-cell RNA-seq and CyTOF, first confirmed the B-cell enrichment, but also decipher a unique immune activity of B cell (increased activity in CXCR4 signaling, cytokine receptor interaction, and chemokine signaling pathways) and a switch in immune activated CXCR3+ memory B cells in responders. The use of single-cell RNAseq and spatial omics in cancer analyze allowed, in this case, to discover unsuspected novel interaction. This comprehensive approach has revealed the structural role of B cell and tertiary lymphoid structures in responses to ICB treatment.

Multi-omics integration for immune characterization has dramatically furthered the identification of biomarkers raising the possibility of personalizing ICB treatment based on the tumor immune constitution.

### Microbiomics as a modulator of the immune environment

The microbiome is the community of microbial species that inhabit a human body (Bhatt et al., 2017). The study of interactions between cancer and the microbiome has gained popularity in the past few years, showing the interplay between cancer features and species colonizing the intestinal flora. The intestinal flora can, indeed, play a role on cancer (Sepich-Poore et al., 2021) by influencing the risk of cancer apparition (Song et al., 2020b) and cancer immune response, it also may be useful for cancer detection (Chen et al., 2021). Theses interactions can lead to mucosal inflammation or systemic metabolic/immune dysregulation and modulate immune responses by altering anti-cancer immunity and response to therapy (Bhatt et al., 2017; Gopalakrishnan et al., 2018; Riquelme et al., 2019; Dohlman et al., 2021; Jackson et al., 2022). In this part, we will show that immunomics and microbiomics data should be integrated into classic omics analysis to master cancer complexity.

There are different sequencing techniques to elucidate the microbiome composition. The first one is the sequencing of the bacterial 16 S ribosomal RNA (rRNA) that is abundant and specific to each species. The 16 S rRNA genes are isolated and amplified from microbiome containing samples and then sequenced for species characterization. The downside of this technique is the need of high accuracy in the primer for amplification (Janda and Abbott, 2007; Pei et al., 2010). A second technique, majorly used in microbiomics, is shotgun sequencing that uses the taxonomic, functional, and genomics profile of the bacteria to deduct the microbiome composition (Quince et al., 2017). The study of microbiome can also be done using classic omics techniques like transcriptomics (metatranscriptomics), proteomics (metaproteomics), and metabolomics used to detect dysregulation of genes, proteins, and metabolites (Franzosa et al., 2014; Bikel et al., 2015; Singhal et al., 2015; Daliri et al., 2017).

Lately, in the aim of cancer treatment, the study of microbiome allowed significant discoveries. It was first stated that antibiotics can disrupt the activity of immunotherapy inducing loss of response to immune checkpoint blockade (ICB). In murine model of melanoma, it was shown that the use of different antibiotics decreases the response of PD-1 treatment compromising its tumor effect (Routy et al., 2018). The same effect was observed in patients with non-small cell lung cancer (NSCLC), renal cell carcinoma (RCC), and urothelial carcinoma. The use of those antibiotics negatively impacted their overall survival and progression-free survival (Derosa et al., 2022) (Routy et al., 2018). This observation suggested that a balanced microbiota is necessary for inducing and maintaining the tumor immune response and prompted further evaluation of microbiome in patients treated with ICB. Microbiome exploration by metagenomics in NSCLC patients treated with ICB confirmed the impact of intestinal flora, showing an enrichment of some species in responders, notably Akkermansia muciniphila (AkM) and Firmicutes, and in non-responder (Prevotella, *Clostridium* species) (Derosa et al., 2018; Routy et al., 2018). A recent work confirmed the role of AkM in response to ICB and introduced the notion relative abundance of bacteria in cancer (Derosa et al., 2022). They demonstrated that a high abundance of AkM can improve the patient outcomes in NSCLC, but if present in high quantity, AkM will be deleterious for ICB response. This raised the hypothesis that restoring microbiota equipoise could restore the response. This was assessed by fecal microbiota transplantation of responder flora in non-responders that was able to shift from unsensitive to sensitive phenotype and restore ICB efficacy. After operating the transplantation, 16 S RNA sequencing revealed that a higher abundance of Veillonellaceae family and poor in *Bifidobacterium bifidum* increased the sensitivity to the treatment (Baruch et al., 2021).

The role of microbiomics for modeling the host-tumor interaction and the immune response to cancer is now established, however, microbiomics has not yet been routinely implemented in multi-omics analysis but is probably a key element of cancer biology. Considering the high plasticity of the microbiome constitution, longitudinal analysis of the flora should be preferred (Turnbaugh et al., 2009; Khoruts et al., 2010; Spencer et al., 2011; Kong et al., 2012). Most importantly, the gut microbiota is highly intricated with the host immune reaction to the tumor warranting its integration in the models of tumor immune environment analysis.

### Improving multi-omics integration in the context of immunomics

As we have shown, combining multiple biological elements of the tumor and of the host environment is an extremely powerful way to anatomize the TiME and to discover new biomarkers. However, there is no standardized method for immunomics and most of the computational integration tools are home made. To improve the generalization of the observation and to accelerate the possibility of patient selection for personalized therapy, standardization of the method is urging.

In that sense, an available R package for computational tool for immuno-oncology biological research (IOBR) has been developed to comprehensively combine and interpret multi-omics data in the context of immuno-oncology (Zeng et al., 2021). IOBR has been built to easily integrate whole exome and RNA sequencing from bulk tumor as well as single-cell RNA sequencing and long non-coding RNA data. It consists of a four-module pipeline with a signature/deconvolution, phenotype, mutation, and model construction. The signature module can identify immune or tumor specific signatures through expression gene profile and deconvolution estimate of the immune infiltrate. The deconvolution part directly integrates CIBERSORT (Newman et al., 2019), TIMER (Li et al., 2020), MCP-Counter (Becht et al., 2016), xCELL (Aran et al., 2017), EPIC (Racle and Gfeller, 2020), and quanTIseq (Finotello et al., 2019). The phenotype module tests a large set of immune and non-immune-related published phenotypes, and the mutation part is able to determine association between different gene alterations and signatures. Finally, this tool has a model construction module that provides robust biomarker identification and model construction from the prior modules. The utilization of standardized ready-to-use package for onco-immunomics analysis is crucial to hasten biomarker discovery and test their inter-cohort reproducibility.

Future directions to fully characterize the immune environment and its dynamic would be to implement microbiomics data to such model and to construct longitudinal models with the introduction of time dependent models (Bodein et al., 2022). The incorporation of spatial annotation and single-cell omics to explore the cancer architecture at the cell level in all its heterogeneity and to complement with functional analysis offers a new venue to elucidate the TiME (Roh et al., 2018; Helmink et al., 2020; Zheng et al., 2021).

## Conclusion

Cancer is a complex and highly heterogenous disease that is yet partially solved. Despite the molecular characterization made possible by the advances of NGS technologies, many of the underlying oncogenic mechanisms remain puzzling. The integration of the multilayers of the omics elements is an avenue to further elucidate cancer biology. In this review, we highlighted the challenges of computational modelization of large multi-omics datasets and we provided some clues for how to overcome these barriers. We presented some innovative bioinformatic tools developed to enlighten the implication of all the interconnected biological elements of cancer and showed how determinant they are to refine the disease subtyping and classification, and to discover biomarkers. Multi-omics integration has also heightened the field of immunomics and microbiomics, and thus has dramatically accelerated the identification of robust biomarkers for ICB efficacy toward the development of tailored immunotherapy.

The constant modernization of the models endows analyses of increasingly larger datasets with a growing number of components. Collaborative effort for high-quality and clinically well-annotated databases, combining all the elements of high-throughput sequencing, is crucial to feed the models. Finally, the standardization of the methods would aid in replicating and confirming the results to increase the global knowledge and, ultimately, improve cancer treatments.
